# Generation of steady entanglement via unilateral qubit driving in bad cavities

**DOI:** 10.1038/s41598-017-17933-7

**Published:** 2017-12-15

**Authors:** Zhao Jin, Shi-Lei Su, Ai-Dong Zhu, Hong-Fu Wang, Li-Tuo Shen, Shou Zhang

**Affiliations:** 1 Department of Physics, Harbin Institute of Technology, Harbin, 150001 China; 2 School of Physical Science Engineering and Key Laboratory of Materials Physics of Ministry of Education of China, Zhengzhou University, Zhengzhou, 450052 China; 3 Department of Physics, College of Science, Yanbian University, Yanji, Jilin 133002 China; 4 Laboratory of Quantum Optics, Department of Physics, Fuzhou University, Fuzhou, 350002 China

## Abstract

We propose a scheme for generating an entangled state for two atoms trapped in two separate cavities coupled to each other. The scheme is based on the competition between the unitary dynamics induced by the classical fields and the collective decays induced by the dissipation of two non-local bosonic modes. In this scheme, only one qubit is driven by external classical fields, whereas the other need not be manipulated via classical driving. This is meaningful for experimental implementation between separate nodes of a quantum network. The steady entanglement can be obtained regardless of the initial state, and the robustness of the scheme against parameter fluctuations is numerically demonstrated. We also give an analytical derivation of the stationary fidelity to enable a discussion of the validity of this regime. Furthermore, based on the dissipative entanglement preparation scheme, we construct a quantum state transfer setup with multiple nodes as a practical application.

## Introduction

Quantum entanglement is an intriguing property of composite systems. The term refers to inseparable correlations that are stronger than all classical counterparts^1,2^. To perform quantum communication safely and perfectly, remote parties are usually required to share a quantum channel of a maximally entangled state. Nevertheless, in real experiments, it is difficult to achieve a perfect quantum channel due to environmental noise. Therefore, in long-distance quantum communication and quantum communication networks, the generation of a steady entanglement between different nodes is significantly challenging^3,4^. The main obstacle in preserving entanglement is dissipation induced by the environment, which is inevitable in the development of quantum science and technology. Generally, entanglement purification^5,6^, together with the use of quantum repeaters^7,8^, is an efficient method for addressing environmental noise. From this perspective, it seems rather conflicting that dissipation can be used as a powerful resource to create entanglement^9–40^ or to realize a spin squeezing state^41,42^. In 1999, Plenio *et al*. and Cabrillo *et al*. presented schemes for engineering entanglement that utilize dissipation^9,10^, which opened up a new chapter for entanglement generation based on dissipative dynamics. Afterward, several schemes were suggested for studying the entanglement in dissipative quantum systems. In particular, Kastoryano, Reitor, and Srensen considered a dissipative scheme for preparing a maximally entangled state of two Λ-type atoms in a high-finesse optical cavity^11^. Their results were better than those obtained via unitary-dynamics-based schemes, as their schemes do not require specifying the initial state or accurately controlling the evolution time. Shen *et al*. generalized this scheme to prepare distributed entanglement via dissipation^12,13^. In addition, Reiter *et al*.^14^ proposed a dissipative scheme in which two transmon qubits can be driven into a steady state in a circuit quantum electrodynamics setup. Recently, the dissipative generation of steady entanglement between two qubits was experimentally achieved using trapped ions^35,36^ and superconducting qubits^37^. Moreover, several interesting schemes concerning the manipulation of quantum states with dissipation dynamics exist, such as the dissipation-induced geometric phase^43^, stimulated Raman adiabatic passage (STIRAP) via dissipative quantum dynamics^44^, and dissipation-assisted quantum state manipulation in cavities^45^.

To perform large-scale quantum computing, the quantum control of separate nodes of a quantum network is indispensable. Coupled cavities are an essential aspect of distributed quantum information processing. One important step is to entangle qubits located in spatially separated resonators. To date, several theoretical^46–53^ and experimental schemes^54^ for generating maximally entangled states have been proposed. Most of these schemes focus on unitary dynamical evolution, in which the inevitable noise can have only detrimental effects, and the system requires precise time control and state initialization.

In this paper, we propose a scheme for preparing and stabilizing a maximally entangled state in coupled cavities by effectively utilizing the unitary dynamics provided by microwave fields and the dissipation originating from cavity decay, where only one atom is driven by two classical fields with precisely chosen frequencies. Our present work has the following features: (i) Our scheme performs well without the need to specify the initial state nor control the evolution time accurately. (ii) This scheme applies unilateral classical driving to only one atom. This is in contrast to ref.^27^, in which both atoms must be driven by three optical lasers. Finally, (iii) our scheme can be generalized to multiple-coupled-cavity models to perform quantum processing tasks such as state transfer between separate nodes. With the currently achievable experiment parameters, the numerical simulation demonstrates that steady-state entanglement with high fidelity, purity, and Clauser-Horne-Shimony-Holt (CHSH) correction can be obtained^55^ and that the scheme is robust against parameter fluctuations. We also give an analytical form of the fidelity to demonstrate the validity of our scheme.

## Results

### Theoretical Model

**Figure 1:**
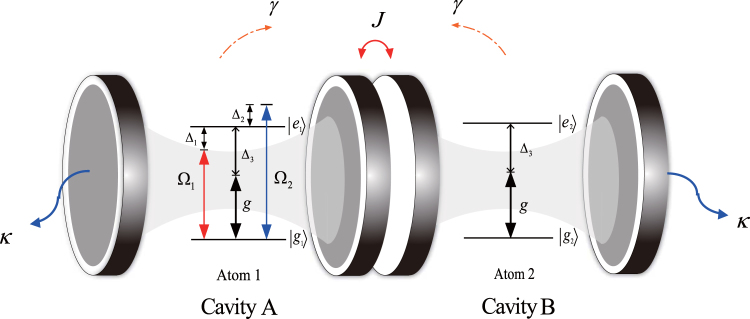
Experimental setup for engineering an entangled steady state for two identical two-level atoms via dissipation in two directly coupled cavities. Two quantized cavity fields are dispersively coupled to the qubits with detuning Δ_3_ and coupling strength *g*. Two off-resonance optical lasers with detuning Δ_*k*_ and Rabi frequency Ω_*k*_ (*k* = 1, 2) simultaneously drive the first atom. *γ* and *κ* are the atomic spontaneous emission rate and the cavity decay rate, respectively.

The coupled-cavity system under consideration is shown in Fig. 1. Two identical two-level atoms 1 and 2 are trapped in two coupled cavities. Each atom has a ground state |*g*〉 and an excited state |*e*〉, with the corresponding energies 0 and *ω*_0_. The atomic transition |*g*_*i*_〉 ↔ |*e*_*i*_〉 (*i* = 1, 2) is dispersively coupled to the cavity mode with the coupling constant *g* and detuning Δ_3_, and the first atom is driven by two off-resonance optical lasers with Rabi frequency Ω_*k*_ and detuning Δ_*k*_ (*k* = 1, 2). Photons can hop between the cavities. The Hamiltonian of the system can be written as (setting *ħ* = 1 throughout this paper) *H* = *H*_*WL*_ + *H*_*CL*_, with

1 $${H}_{WL}=\sum _{i=1,2}\,{\omega }_{0}|{e}_{i}\rangle \langle {e}_{i}|+\sum _{j=A,B}\,{\omega }_{a}{a}_{j}^{\dagger }{a}_{j}+J({a}_{A}^{\dagger }{a}_{B}+{a}_{A}{a}_{B}^{\dagger })+[g({S}_{1}^{\dagger }{a}_{A}+{S}_{2}^{\dagger }{a}_{B})+{\rm{H}}.{\rm{c}}.],$$

2 $${H}_{CL}=\sum _{k=1,2}\,{{\rm{\Omega }}}_{k}{e}^{-i{\omega }_{k}t}{S}_{1}^{\dagger }+{\rm{H}}.{\rm{c}}.,$$

where ${S}_{i}^{\dagger }=|{e}_{i}\rangle \langle {g}_{i}|$ (*i* = 1, 2); |*e*_*i*_〉 and |*g*_*i*_〉 are the excited and ground states of the *i*th qubit, respectively; *a*_*j*_ and ${a}_{j}^{\dagger }$ denote the annihilation and creation operators for the optical mode of cavity *j*, respectively; Ω_*k*_ and *ω*_*k*_ represent the amplitude and frequency of the *k*th driving field, respectively; *ω*_*a*_ denotes the frequency of the cavity mode; and *J* is the photon-hopping strength, which describes the coupling between two cavities. To simplify the dynamics of the system, we introduce the non-local bosonic modes

3 $$\begin{array}{l}{c}_{1}=\frac{\sqrt{2}}{2}({a}_{A}+{a}_{B}),\\ {c}_{2}=\frac{\sqrt{2}}{2}({a}_{A}-{a}_{B}).\end{array}$$

These two modes are not coupled with each other and are linearly related to the field modes of the two cavities. In terms of the new operators, the Hamiltonian *H*_*WL*_ can be rewritten as

4 $${H}_{WL}=\sum _{i=1,2}\,{\omega }_{0}|{e}_{i}\rangle \langle {e}_{i}|+({\omega }_{a}+J){c}_{1}^{\dagger }{c}_{1}+({\omega }_{a}-J){c}_{2}^{\dagger }{c}_{2}+\frac{g}{\sqrt{2}}[{S}_{1}^{\dagger }({c}_{1}+{c}_{2})+{S}_{2}^{\dagger }({c}_{1}-{c}_{2})+{\rm{H}}.{\rm{c}}.].$$

The Hamiltonian *H*_*WL*_ describes the coupling of the two atoms to the delocalized field modes *c*_1_ and *c*_2_ with frequencies *ω*_*a*_ + *J* and *ω*_*a*_ − *J*. In the interaction picture with respect to ${H}_{0}={\sum }_{i=1,2}{\omega }_{0}|{e}_{i}\rangle \langle {e}_{i}|+({\omega }_{a}+J){c}_{1}^{\dagger }{c}_{1}+({\omega }_{a}-J){c}_{2}^{\dagger }{c}_{2}$, we obtain the interaction Hamiltonian

5 $${H}_{WL}^{I}=\frac{\sqrt{2}}{2}g{e}^{i{{\rm{\Delta }}}_{3}t}[({e}^{-iJt}{c}_{1}+{e}^{iJt}{c}_{2}){S}_{1}^{\dagger }+({e}^{-iJt}{c}_{1}-{e}^{iJt}{c}_{2}){S}_{2}^{\dagger }+{\rm{H}}.{\rm{c}}.],$$

in which Δ_3_ = *ω*_0_ − *ω*_*a*_. Meanwhile, bosonic mode *c*_1_ is resonant with the two qubits, and bosonic mode *c*_2_ is largely dispersive with the two qubits under the conditions of *J* = Δ_3_ and ${{\rm{\Delta }}}_{3}+J\gg g$. Therefore, after discarding the rapidly oscillating terms, the interaction Hamiltonian reduces to

6 $${H}_{WL}^{I^{\prime} }=\frac{\sqrt{2}}{2}g({S}_{1}^{\dagger }+{S}_{2}^{\dagger }){c}_{1}+{\rm{H}}.{\rm{c}}.$$

The dynamics of an open dissipative system in Lindblad form is described by the master equation

7 $$\dot{\hat{\rho }}(t)=i[\hat{\rho }(t),H]+\frac{1}{2}\,\sum _{j}\,[2{\hat{L}}_{j}\hat{\rho }(t){\hat{L}}_{j}^{\dagger }-({\hat{L}}_{j}^{\dagger }{\hat{L}}_{j}\hat{\rho }(t)+\hat{\rho }(t){\hat{L}}_{j}^{\dagger }{\hat{L}}_{j})],$$

where *H* represents the original Hamiltonian of the whole system, as shown in Eqs (1) and (2), and the Lindblad superoperator is defined as ${\hat{L}}_{j}$. Specifically, in the current scheme, the Lindblad operators can be expressed as ${\hat{L}}_{\kappa i}=\sqrt{\kappa }{a}_{i}$ (*i* = *A*, *B*), which describes the dissipation induced by the leakage of cavity A and cavity B, and ${\hat{L}}_{\gamma n}=\sqrt{\gamma }|{g}_{n}\rangle \langle {e}_{n}|$ (*n* = 1, 2), which describes the dissipation induced by the spontaneous emission of the two atoms. *κ* and *γ* are the leakage rate of the cavities and the atomic spontaneous emission rate, respectively.

### Preparation of the steady entanglement

**Figure 2:**
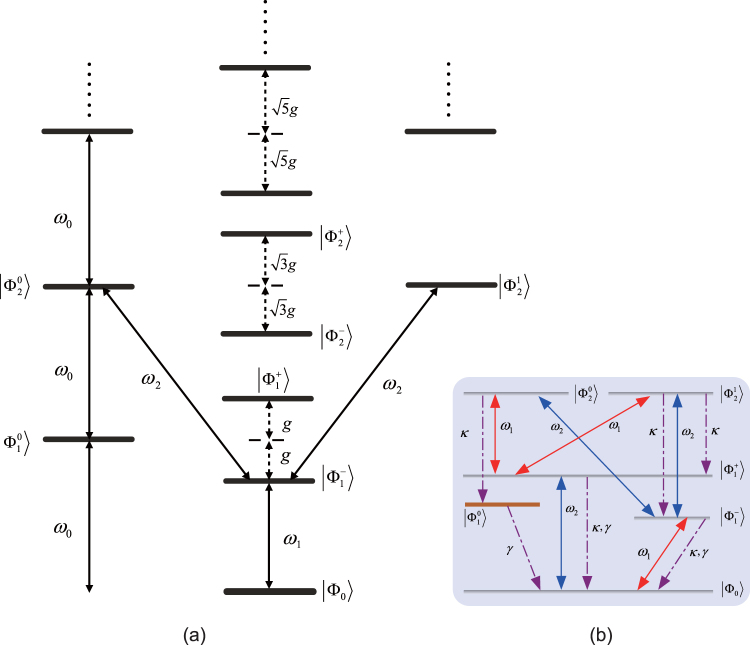
(**a**) Level configuration in the dressed state picture. (**b**) Processes for producing and stabilizing Bell states. The interaction between the system and the environment is characterized by photon loss and atomic spontaneous emission, with rates *κ* and *γ*, respectively.

We transform the Hamiltonian ${H}_{WL}^{I^{\prime} }$ in Eq. (6) back into the original picture. The spectroscopy of the system is well described by the dressed states, i.e., the eigenstates of the Hamiltonian ${H}_{0}+{H}_{WL}^{I^{\prime} }$, as shown in Fig. 2(a). Note that the excitation number of the total system ${N}_{e}={\sum }_{i=1,2}(|{e}_{i}\rangle \langle {e}_{i}|+{c}_{i}^{\dagger }{c}_{i})$ commutes with *H*_0_ and ${H}_{WL}^{I^{\prime} }$; the excitation number is thus conserved under the control of these two Hamiltonians. Nevertheless, the Hamiltonian *H*_*CL*_ and Lindblad superoperator ${\hat{L}}_{j}$ will change the excitation number because these two operators do not commute with the excitation number operator. By choosing suitable driving field frequencies, the transitions to the Hilbert subspaces with three or more excitations are off-resonance with the two classical fields due to the unequal spacings of the energy levels of the dressed states. When the Rabi frequencies of the classical fields are substantially smaller than the atom-cavity mode coupling strength, i.e., ${{\rm{\Omega }}}_{1},\,{{\rm{\Omega }}}_{2}\ll g$, populations of the dressed states with more than two excitations can be neglected. Therefore, we can restrict our analysis to the physical mechanism of dissipative preparation in the Hilbert subspace up to two excitations. We denote the dressed state of the coupled system as |*A*, *B*〉 |*C*, *D*〉, where the first and second ket represent the state of the two atoms and of the two delocalized bosonic modes, respectively. *A* ∈ {*e*_1_, *g*_1_}, *B* ∈ {*e*_2_, *g*_2_} and *C*(*D*) ∈ {*m*}, with *m* being the positive integer used to denote the photon number. The dressed states $|{{\rm{\Phi }}}_{{N}_{e}}\rangle$ with the corresponding eigenvalue ${E}_{{N}_{e}}$ within the excitation subspace *N*_*e*_ can be expressed as follows: the ground state |Φ_0_〉 = |*g*_1_, *g*_2_〉 |0, 0〉 (*E*_0_ = 0); the one-excitation states $|{{\rm{\Phi }}}_{1}^{0}\rangle =|{\phi }_{-}\rangle \mathrm{|0},0\rangle$ (${E}_{1}^{0}={\omega }_{0}$) and $|{{\rm{\Phi }}}_{1}^{\pm }\rangle =\frac{1}{\sqrt{2}}[|{\phi }_{+}\rangle \mathrm{|0},0\rangle \pm |{g}_{1},{g}_{2}\rangle \mathrm{|1},0\rangle ]$ (${E}_{1}^{\pm }={\omega }_{0}\pm g$), where $|{\phi }_{\pm }\rangle =1/\sqrt{2}(|{e}_{1},{g}_{2}\rangle \pm |{g}_{1},{e}_{2}\rangle )$ and $|{{\rm{\Phi }}}_{1}^{0}\rangle$ is the maximal entanglement to be prepared for the two atoms; and the two-excitation states $|{{\rm{\Phi }}}_{2}^{0}\rangle =|{\phi }_{-}\rangle \mathrm{|1},0\rangle$ (${E}_{2}^{0}=2{\omega }_{0}$), $|{{\rm{\Phi }}}_{2}^{1}\rangle =\frac{1}{\sqrt{3}}(|{g}_{1},{g}_{2}\rangle \mathrm{|2,0}\rangle -\sqrt{2}|{e}_{1},{e}_{2}\rangle )\mathrm{|0},0\rangle$ (${E}_{2}^{1}=2{\omega }_{0}$), and $|{{\rm{\Phi }}}_{2}^{\pm }\rangle =\frac{1}{\sqrt{2}}[(|{\phi }_{+}\rangle \mathrm{)|1},0\rangle \pm$ $\frac{1}{\sqrt{3}}(\sqrt{2}|{g}_{1},{g}_{2}\rangle \mathrm{|2},0\rangle$ $+|{e}_{1},{e}_{2}\rangle \mathrm{|0},0\rangle )]$ $({E}_{2}^{\pm }=2{\omega }_{0}\pm \sqrt{3}g)$. These dressed states are the eigenstates of the Hamiltonian ${H}_{0}+{H}_{WL}^{I^{\prime} }$, which can be considered as a set of approximately complete bases. Under the dressed state picture, the Hamiltonian *H*_*CL*_ can be rewritten as

8 $$\begin{array}{rcl}{H}_{CL} & = & \sum _{k=1,2}\,{{\rm{\Omega }}}_{k}{e}^{-i{\omega }_{k}t}[\frac{1}{2}(|{{\rm{\Phi }}}_{1}^{-}\rangle +|{{\rm{\Phi }}}_{1}^{+}\rangle )\langle {{\rm{\Phi }}}_{0}|+\frac{1}{\sqrt{2}}|{{\rm{\Phi }}}_{1}^{0}\rangle \langle {{\rm{\Phi }}}_{0}|\\  &  & +\frac{1}{2\sqrt{3}}(|{{\rm{\Phi }}}_{2}^{-}\rangle -|{{\rm{\Phi }}}_{2}^{+}\rangle )\langle {{\rm{\Phi }}}_{1}^{0}|+\frac{1}{\sqrt{3}}|{{\rm{\Phi }}}_{2}^{1}\rangle \langle {{\rm{\Phi }}}_{1}^{0}|\\  &  & +(\frac{1}{2\sqrt{6}}+\frac{1}{2\sqrt{2}})|{{\rm{\Phi }}}_{2}^{+}\rangle \langle {{\rm{\Phi }}}_{1}^{+}|+\frac{1}{2}|{{\rm{\Phi }}}_{2}^{0}\rangle \langle {{\rm{\Phi }}}_{1}^{+}|\\  &  & +(\frac{1}{2\sqrt{2}}-\frac{1}{2\sqrt{6}})|{{\rm{\Phi }}}_{2}^{-}\rangle \langle {{\rm{\Phi }}}_{1}^{+}|-\frac{1}{\sqrt{6}}|{{\rm{\Phi }}}_{2}^{1}\rangle \langle {{\rm{\Phi }}}_{1}^{+}|\\  &  & +(\frac{1}{2\sqrt{6}}-\frac{1}{2\sqrt{2}})|{{\rm{\Phi }}}_{2}^{+}\rangle \langle {{\rm{\Phi }}}_{1}^{-}|-\frac{1}{2}|{{\rm{\Phi }}}_{2}^{0}\rangle \langle {{\rm{\Phi }}}_{1}^{-}|\\  &  & -(\frac{1}{2\sqrt{2}}+\frac{1}{2\sqrt{6}})|{{\rm{\Phi }}}_{2}^{-}\rangle \langle {{\rm{\Phi }}}_{1}^{-}|-\frac{1}{\sqrt{6}}|{{\rm{\Phi }}}_{2}^{1}\rangle \langle {{\rm{\Phi }}}_{1}^{-}|+{\rm{H}}.{\rm{c}}.]\mathrm{.}\end{array}$$

We proceed to the interaction picture with respect to the Hamiltonian ${H}_{0}+{H}_{WL}^{I^{\prime} }$ expressed by the eigenvectors and eigenvalues in the zero-, one- and two-excitation subspaces. Eq. (8) can be rewritten as

9 $$\begin{array}{rcl}{H}_{CL}^{I} & = & \sum _{k=\mathrm{1,2}}\,{{\rm{\Omega }}}_{k}[\frac{1}{2}({e}^{i({{\rm{\Delta }}}_{k}-g)t}|{{\rm{\Phi }}}_{1}^{-}\rangle \langle {{\rm{\Phi }}}_{0}|+{e}^{i({{\rm{\Delta }}}_{k}+g)t}|{{\rm{\Phi }}}_{1}^{+}\rangle \langle {{\rm{\Phi }}}_{0}|)\\  &  & +{e}^{i{{\rm{\Delta }}}_{k}t}(\frac{1}{\sqrt{2}}|{{\rm{\Phi }}}_{1}^{0}\rangle \langle {{\rm{\Phi }}}_{0}|+\frac{1}{\sqrt{3}}|{{\rm{\Phi }}}_{2}^{1}\rangle \langle {{\rm{\Phi }}}_{1}^{0}|)\\  &  & +\frac{1}{2\sqrt{3}}({e}^{i({{\rm{\Delta }}}_{k}-\sqrt{3}g)t}|{{\rm{\Phi }}}_{2}^{-}\rangle \langle {{\rm{\Phi }}}_{1}^{0}|-{e}^{i({{\rm{\Delta }}}_{k}+\sqrt{3}g)t}|{{\rm{\Phi }}}_{2}^{+}\rangle \langle {{\rm{\Phi }}}_{1}^{0}|)\\  &  & +(\frac{1}{2\sqrt{6}}+\frac{1}{2\sqrt{2}}){e}^{i[{{\rm{\Delta }}}_{k}+(\sqrt{3}-\mathrm{1)}g]t}|{{\rm{\Phi }}}_{2}^{+}\rangle \langle {{\rm{\Phi }}}_{1}^{+}|\\  &  & +(\frac{1}{2\sqrt{2}}-\frac{1}{2\sqrt{6}}){e}^{i[{{\rm{\Delta }}}_{k}-(\sqrt{3}+\mathrm{1)}g]t}|{{\rm{\Phi }}}_{2}^{-}\rangle \langle {{\rm{\Phi }}}_{1}^{+}|\\  &  & +{e}^{i({{\rm{\Delta }}}_{k}-g)t}(\frac{1}{2}|{{\rm{\Phi }}}_{2}^{0}\rangle \langle {{\rm{\Phi }}}_{1}^{+}|-\frac{1}{\sqrt{6}}|{{\rm{\Phi }}}_{2}^{1}\rangle \langle {{\rm{\Phi }}}_{1}^{+}|)\\  &  & +(\frac{1}{2\sqrt{6}}-\frac{1}{2\sqrt{2}}){e}^{i[{{\rm{\Delta }}}_{k}+(\sqrt{3}+\mathrm{1)}g]t}|{{\rm{\Phi }}}_{2}^{+}\rangle \langle {{\rm{\Phi }}}_{1}^{-}|\\  &  & -(\frac{1}{2\sqrt{2}}+\frac{1}{2\sqrt{6}}){e}^{i[{{\rm{\Delta }}}_{k}-(\sqrt{3}-\mathrm{1)}g]t}|{{\rm{\Phi }}}_{2}^{-}\rangle \langle {{\rm{\Phi }}}_{1}^{-}|\\  &  & -{e}^{i({{\rm{\Delta }}}_{k}+g)t}(\frac{1}{2}|{{\rm{\Phi }}}_{2}^{0}\rangle \langle {{\rm{\Phi }}}_{1}^{-}|+\frac{1}{\sqrt{6}}|{{\rm{\Phi }}}_{2}^{1}\rangle \langle {{\rm{\Phi }}}_{1}^{-}|)+{\rm{H}}.{\rm{c}}.]\mathrm{.}\end{array}$$

By setting the detuning Δ_*k*_ = *ω*_0_ − *ω*_*k*_ equals to −(−1)^*k*^ *g* (*k* = 1, 2), the driving field Ω_1_ resonantly drives the transitions $|{{\rm{\Phi }}}_{0}\rangle \leftrightarrow |{{\rm{\Phi }}}_{1}^{-}\rangle$, $|{{\rm{\Phi }}}_{1}^{+}\rangle \leftrightarrow |{{\rm{\Phi }}}_{2}^{0}\rangle$ and $|{{\rm{\Phi }}}_{1}^{+}\rangle \leftrightarrow |{{\rm{\Phi }}}_{2}^{1}\rangle$; Ω_2_ resonantly drives $|{{\rm{\Phi }}}_{0}\rangle \leftrightarrow |{{\rm{\Phi }}}_{1}^{+}\rangle$, $|{{\rm{\Phi }}}_{1}^{-}\rangle \leftrightarrow |{{\rm{\Phi }}}_{2}^{0}\rangle$ and $|{{\rm{\Phi }}}_{1}^{-}\rangle \leftrightarrow |{{\rm{\Phi }}}_{2}^{1}\rangle$; and all other transitions between any two arbitrary dressed states are largely detuned. Under the weak excitation condition, i.e., ${{\rm{\Omega }}}_{k}\ll g$, using the rotating wave approximation, we can adiabatically eliminate the non-resonance coupling terms. The Hamiltonian ${H}_{CL}^{I}$ reduces to

10 $$\begin{array}{rcl}{H}_{CL}^{I^{\prime} } & = & {{\rm{\Omega }}}_{1}(\frac{1}{2}|{{\rm{\Phi }}}_{1}^{-}\rangle \langle {{\rm{\Phi }}}_{0}|+\frac{1}{2}|{{\rm{\Phi }}}_{2}^{0}\rangle \langle {{\rm{\Phi }}}_{1}^{+}|-\frac{1}{\sqrt{6}}|{{\rm{\Phi }}}_{2}^{1}\rangle \langle {{\rm{\Phi }}}_{1}^{+}|)\\  &  & \times {{\rm{\Omega }}}_{2}(\frac{1}{2}|{{\rm{\Phi }}}_{1}^{+}\rangle \langle {{\rm{\Phi }}}_{0}|-\frac{1}{2}|{{\rm{\Phi }}}_{2}^{0}\rangle \langle {{\rm{\Phi }}}_{1}^{-}|-\frac{1}{\sqrt{6}}|{{\rm{\Phi }}}_{2}^{1}\rangle \langle {{\rm{\Phi }}}_{1}^{-}|)+{\rm{H}}.{\rm{c}}.\end{array}$$

From the above equation, we can see that the target state $|{{\rm{\Phi }}}_{0}^{1}\rangle$ is decoupled from the Hamiltonian ${H}_{CL}^{I\,^{\prime} }$. Hence, we can derive $-i[{H}_{CL}^{I^{\prime} },|{{\rm{\Phi }}}_{1}^{0}\rangle \langle {{\rm{\Phi }}}_{1}^{0}|]=0$. To generate the required Bell state, here, we require the atomic spontaneous emission to be much slower than other dynamical processes, i.e., $\gamma \ll {{\rm{\Omega }}}_{1},{{\rm{\Omega }}}_{2},\kappa$, so that it can be ignored, which guarantees that any transitioning away from the target state is strongly suppressed. Since $|{{\rm{\Phi }}}_{0}^{1}\rangle =|{\phi }_{-}\rangle \otimes \mathrm{|00}\rangle$ is a Kronecker product state of the Bell state and the two delocalized vacuum bosonic modes, it is unaffected by the photon decay, i.e., $\frac{1}{2}{\sum }_{i}\,[2{\hat{L}}_{\kappa i}|{{\rm{\Phi }}}_{1}^{0}\rangle \langle {{\rm{\Phi }}}_{1}^{0}|{\hat{L}}_{\kappa i}^{\dagger }-({\hat{L}}_{\kappa i}^{\dagger }{\hat{L}}_{\kappa i}|{{\rm{\Phi }}}_{1}^{0}\rangle \langle {{\rm{\Phi }}}_{1}^{0}|+|{{\rm{\Phi }}}_{1}^{0}\rangle \langle {{\rm{\Phi }}}_{1}^{0}|{\hat{L}}_{\kappa i}^{\dagger }{\hat{L}}_{\kappa i})]=0$ (*i* = *A*, *B*). Therefore, the transitions associated with the target state $|{{\rm{\Phi }}}_{0}^{1}\rangle$ are hardly affected by classical drivings and cavity decay, so that it is a steady state.

The processes for producing and stabilizing the Bell state $|{{\rm{\Phi }}}_{1}^{0}\rangle$ are shown in Fig. 2(b). The initial system state |Φ_0_〉 is driven by the classical field Ω_1_ (Ω_2_) to the one-excitation dressed state $|{{\rm{\Phi }}}_{1}^{-}\rangle$ ($|{{\rm{\Phi }}}_{1}^{+}\rangle$) and then to $|{{\rm{\Phi }}}_{2}^{0}\rangle$ and $|{{\rm{\Phi }}}_{2}^{1}\rangle$ by the classical field Ω_2_ (Ω_1_). The photon loss in the cavities results in the decaying channel $|{{\rm{\Phi }}}_{2}^{0}\rangle \to |{{\rm{\Phi }}}_{1}^{0}\rangle =|{\phi }_{+}\rangle \mathrm{|0},0\rangle$. On the other hand, the state $|{{\rm{\Phi }}}_{2}^{1}\rangle$ decays to the one-excitation dressed state $|{{\rm{\Phi }}}_{1}^{\pm }\rangle$, being repumped by the classical fields Ω_1_ and Ω_2_ to $|{{\rm{\Phi }}}_{2}^{0}\rangle$ and $|{{\rm{\Phi }}}_{2}^{1}\rangle$; then, with the coherent driving and dissipation processes continuing, the population of the dressed state $|{{\rm{\Phi }}}_{2}^{1}\rangle$ gradually declines until the entire qubit population is driven to the Bell state $|{{\rm{\Phi }}}_{1}^{0}\rangle$.

## Discussion

**Figure 3:**
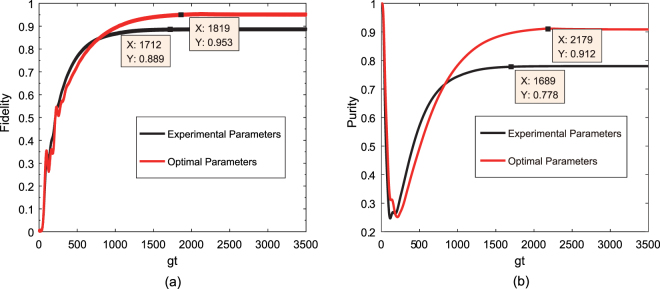
Fidelity (**a**) and purity (**b**) of the target state $|{{\rm{\Phi }}}_{1}^{0}\rangle$ versus the dimensionless parameter *gt* for the initial state |Φ_0_〉 with the optimized Rabi frequencies of drivings Ω_1_ = 0.037 *g* and Ω_2_ = 0.0775 *g* and a cavity-cavity hopping strength *J* = 20 *g*. The chosen experimental parameters are *γ* = 2.72 × 10^−4^ *g* and *κ* = 2.8 × 10^−2^ *g*. In addition, the chosen optimized parameters are *γ* = 1.75 × 10^−5^ *g* and *κ* = 1.6 × 10^−2^ *g*.

To demonstrate the feasibility of our Bell-state stabilization mechanism, we assess the performance of our scheme by numerically solving the Lindblad master equation. Under the condition that the atomic spontaneous emission rate is much slower than other dynamical processes, i.e., $\gamma \ll \kappa ,\,{{\rm{\Omega }}}_{1},\,{{\rm{\Omega }}}_{2}$, we choose the following parameters from a recent circuit QED experiment^37^: $\chi /2\pi \simeq 6$ MHz, $\kappa /2\pi \simeq 1.7$ MHz, ${T}_{1}\simeq 9$ μs, where *T*_1_ is the qubit energy relaxation time, and *χ* = *g* ^2^/Δ, with Δ being the qubit-resonator detuning. It is reasonable to set Δ = 10 *g* in this dispersive interaction system, yielding $g/2\pi \simeq 60\,MHz$, $\kappa \simeq 2.8\times {10}^{-2}\,g$, and $\gamma \simeq 2.72\times {10}^{-4}\,g$. We have taken the optimized Rabi frequencies of the drivings Ω_1_ = 0.037 *g* and Ω_2_ = 0.0775 *g* and the cavity-cavity hopping strength *J* = 20 *g*. In Fig. 3(a), we plot the evolutions of the fidelity $F(t)={\rm{Tr}}[(|{\phi }_{+}\rangle \langle {\phi }_{+}|\otimes {I}_{c})\hat{\rho }(t\to \infty )]$ with experimental and optimized parameters. The results show that the desired state can be prepared with a fidelity greater than 88% when the evolution time $t\simeq 1712/g$. By taking *γ* = 1.75 × 10^−5^ *g* and *κ* = 1.6 × 10^−2^ *g*, the optimal fidelity for the target state is approximately 95.3% when the evolution time $t\simeq 1819/g$. Dissipative processes generally lead to the production of mixed states; therefore, we introduce purity to characterize the mixture degree of the target state. With the above two sets of parameters, in Fig. 3(b), the purity $P(t)={\rm{Tr}}[\hat{\rho }{(t)}^{2}]$ of the target state is plotted as a function of the evolution time. The target state can be stabilized using an experimental purity of approximately 83% and an optimal purity of approximately 94%. Because the initial state of the system is |Φ_0_〉, the purity is precisely unity. In addition, note that the purity curve exhibits a valley in the regime 0 < *t* < 500/*g*. This valley occurs because the coherent driving is dominant in the early stages of evolution, thus leading the system to be in a mixture of a variety of quantum states. With increasing evolution time, the competition between the coherent driving and dissipation drives the system to a dynamic equilibrium, which is a mixture of specific dressed states.

**Figure 4:**
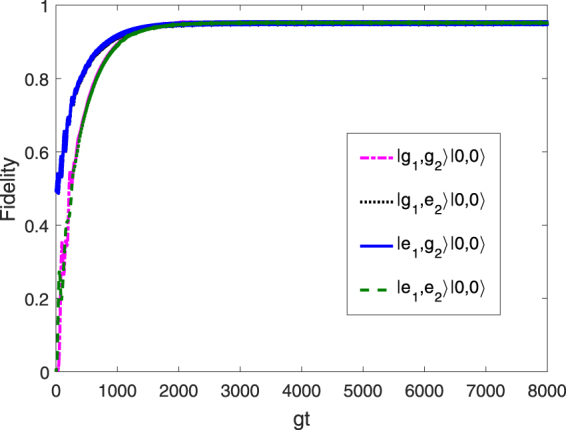
Fidelity of $|{{\rm{\Phi }}}_{1}^{0}\rangle$, plotted versus *gt* for four different initial states |*g*_1_, *g*_2_〉 |0, 0〉, |*g*_1_, *e*_2_〉 |0, 0〉, |*e*_1_, *g*_2_〉 |0, 0〉, and |*e*_1_, *e*_2_〉 |0, 0〉 with the optimized parameters Ω_1_ = 0.037 *g*, Ω_2_ = 0.0775 *g*, *J* = 20 *g*, *γ* = 1.75 × 10^−5^ *g*, and *κ* = 1.6 × 10^−2^ *g*.

To inspect the uniqueness of target state $|{{\rm{\Phi }}}_{0}^{1}\rangle$ numerically, in Fig. 4, we show the behavior of the fidelity with respect to the target state for four different initial states |*g*_1_, *g*_2_〉 |0, 0〉, |*g*_1_, *e*_2_〉 |0, 0〉, |*g*_1_, *e*_2_〉 |0, 0〉, and |*e*_1_, *e*_2_〉 |0, 0〉. We see that for the optimized parameters Ω_1_ = 0.037 *g*, Ω_2_ = 0.0775 *g*, *J* = 20 *g*, *γ* = 1.75 × 10^−5^ *g*, and *κ* = 1.6 × 10^−2^ *g*, any initial state can be driven to the target state asymptotically. This can be explained as follows. Consider the initial state |*e*_1_, *g*_2_〉 |0, 0〉 or |*g*_1_, *e*_2_〉 |0, 0〉, which can be regarded as a superposition of the one-excitation dressed states $|{{\rm{\Phi }}}_{1}^{0}\rangle$ and $|{{\rm{\Phi }}}_{1}^{\pm }\rangle$. As has been shown, due to the coherent driving and dissipation process, the populations of $|{{\rm{\Phi }}}_{1}^{\pm }\rangle$ are ultimately transferred to the target steady state when the evolution time $t\simeq 1791/g$. Similarly, if the initial state is |*e*_1_, *e*_2_〉 |0, 0〉, which is a specific superposition of the dressed states $|{{\rm{\Phi }}}_{2}^{1}\rangle$ and $|{{\rm{\Phi }}}_{2}^{\pm }\rangle$, the state should evolve to the states |*ϕ*_−_〉 |1, 0〉 and |*g*_1_, *g*_2_〉 |2, 0〉 induced by the coupling between the qubits and the collective photon modes. These two states continuously decay to |*ϕ*_−_〉 |0, 0〉 and |*g*_1_, *g*_2_〉 |1, 0〉 due to collective photon losses. The state |*g*_1_, *g*_2_〉 |1, 0〉, which can be expanded using the dressed states $|{{\rm{\Phi }}}_{1}^{+}\rangle$ and $|{{\rm{\Phi }}}_{1}^{-}\rangle$, will undoubtedly be transformed into the steady entanglement state when the evolution time $t\simeq 1819/g$. Therefore, the generation of the target steady state is independent of the initial state, and every choice can lead to identical population for the target state.

**Figure 5:**
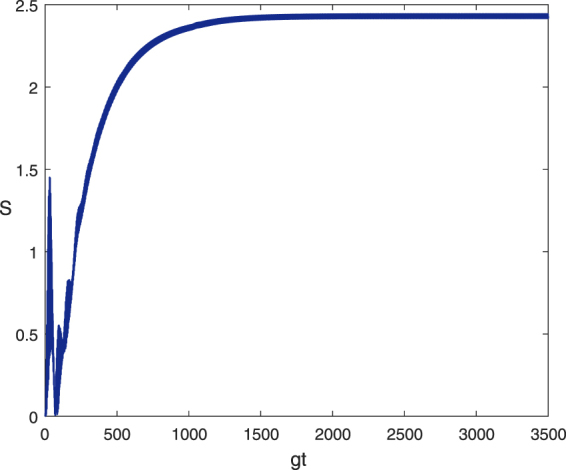
CHSH correlation as a function of *gt* for the preset parameters Ω_1_ = 0.037 *g*, Ω_2_ = 0.0775 *g*, and *J* = 20 *g*. The chosen experimental dissipative factors are *γ* = 2.72 × 10^−4^ *g* and *κ* = 2.8 × 10^−2^ *g*.

The CHSH correlation *S*(*t*) is defined as^55^

11 $$S(t)={\rm{Tr}}[({O}_{{\rm{CHSH}}})\rho (t)],$$

with

12 $$\begin{array}{rcl}{O}_{{\rm{CHSH}}} & = & {\sigma }_{y\mathrm{,1}}\otimes \frac{-{\sigma }_{y\mathrm{,2}}-{\sigma }_{x\mathrm{,2}}}{\sqrt{2}}+{\sigma }_{x\mathrm{,1}}\otimes \frac{-{\sigma }_{y\mathrm{,2}}-{\sigma }_{x\mathrm{,2}}}{\sqrt{2}}\\  &  & +{\sigma }_{x\mathrm{,1}}\otimes \frac{{\sigma }_{y\mathrm{,2}}-{\sigma }_{x\mathrm{,2}}}{\sqrt{2}}-{\sigma }_{y\mathrm{,1}}\otimes \frac{{\sigma }_{y\mathrm{,2}}-{\sigma }_{x\mathrm{,2}}}{\sqrt{2}},\end{array}$$

where *σ*_*x*,1_(*σ*_*x*,2_) and *σ*_*y*,1_(*σ*_*y*,2_) are the Pauli operators of atom 1(2). With the above experimental parameters, the CHSH correlation versus the evolution time is plotted in Fig. 5, from which one can observe a value of approximately 2.45, clearly exceeding the maximum value of 2 allowed by local hidden variable theories.

**Figure 6:**
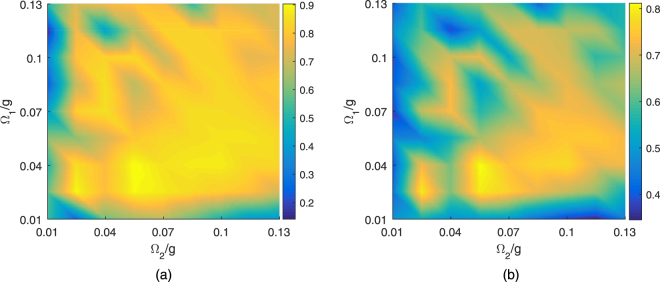
Fidelity (**a**) and purity (**b**) of the target state as a function of the parameters Ω_1_/*g* and Ω_2_/*g* with the initial state |Φ_0_〉 at the time 4 × 10^3^/*g*. The chosen parameters are Ω_1_ = 0.037 *g*, Ω_2_ = 0.0775 *g*, *J* = 20 *g*, *γ* = 2.72 × 10^−4^ *g*, and *κ* = 2.8 × 10^−2^ *g*.

When referring to experimental implementation, it is possible that some system parameters may be unable to maintain their preset values as expected. In Fig. 6(a,b), the fidelity and purity are plotted as functions of the Rabi frequencies Ω_1_ and Ω_2_, respectively, for the given dissipative factors *κ* and *γ*. The results show that the fidelity and purity are higher than 80% and 70%, respectively, within a wide range of Rabi frequencies, demonstrating that the scheme is insensitive to deviations of the control parameters Ω_1_ and Ω_2_.

**Figure 7:**
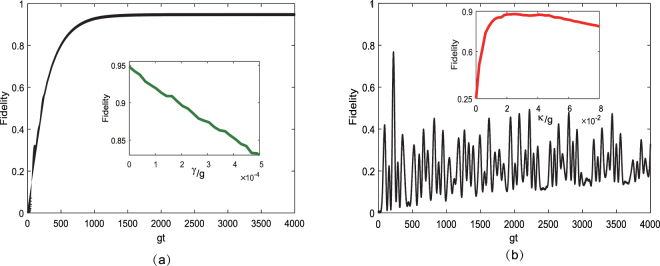
(**a**,**b**) Fidelity of the steady state $|{{\rm{\Phi }}}_{1}^{0}\rangle$ versus *gt* with the initial state |Φ_0_〉. The chosen parameters are Ω_1_ = 0.037 *g*, Ω_2_ = 0.0775 *g*, and *J* = 20 *g*. In (**a**), the selected dissipative factors are *γ* = 0 and *κ* = 2.8 × 10^−2^ *g*. The inset of (a) shows the fidelity of the steady state as a function of the atomic spontaneous rate *γ* for the dissipative factors *κ* = 2.8 × 10^−2^ *g*. *γ* varies from 0 to 5 × 10^−4^ *g* at time 4 × 10^3^/*g*. In (**b**), the selected dissipative factors are *κ* = 0 and *γ* = 2.72 × 10^−4^ *g*. The inset of (**b**) shows the fidelity of the steady state as a function of the cavity decay rate *κ* with the dissipative factor *γ* = 2.72 × 10^−4^ *g*. *κ* varies from 0 to 8 × 10^−2^ *g* at time 4 × 10^3^/*g*.

To clearly observe the role of each dissipative factor, we first consider the system without spontaneous emission, and then we consider it without cavity decay. In Fig. 7(a), we plot the fidelity of the target state $|{{\rm{\Phi }}}_{1}^{0}\rangle$ by taking the atomic spontaneous emission rate as *γ* = 0 and the cavity decay rate as *κ* = 2.8 × 10^−2^ *g*, which shows that the desired state can be prepared with a fidelity of 94.5% and that the time for the system to reach the steady state is approximately *t* = 1413/*g*. This result is approximately 5% higher than that of the situation for *γ* = 2.72 × 10^−4^ *g* in Fig. 3(a). The inset in Fig. 7(a) shows that when the system is stabilized, i.e., when the evolution time is fixed at *t* = 4 × 10^3^/*g*, the fidelity decreases with increasing *γ*. This result occurs because as *γ* increases, the transition rate from the target steady state to the ground state |Φ_0_〉 becomes stronger such that the fidelity decreases. This proves that the ideal entanglement generation scheme requires the atomic spontaneous emission rate *γ* = 0. In Fig. 7(b), we plot the fidelity of the target state by taking the cavity decay rate as *κ* = 0 and the atomic spontaneous emission rate as *γ* = 2.72 × 10^−4^ *g*. The results show that when the values of the cavity decay rate are set to zero, the scheme cannot succeed. In the inset of Fig. 7(b), we choose the evolution time as *t* = 4 × 10^3^/*g* and plot the fidelity of the presented scheme with varying cavity decay rate *κ*. As *κ* increases, the fidelity clearly increases. Nevertheless, further increases in the cavity decay will have an adverse effect on the unitary dynamics and thus decrease the overall performance of the scheme. Therefore, the results provide further verification that the ideal entanglement generation scheme can be achieved based on cavity decay.

**Figure 8:**
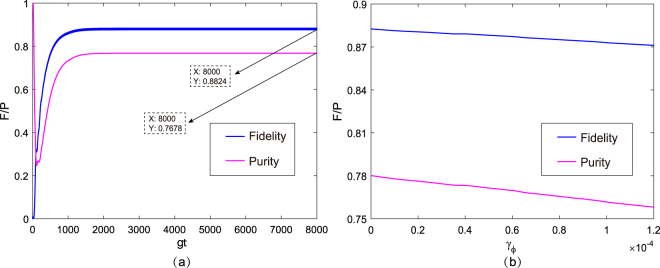
(**a**) Fidelity and purity of the steady state $|{{\rm{\Phi }}}_{1}^{0}\rangle$ as a function of *gt* with the initial state |Φ_0_〉 when considering the effect of pure dephasing, where the selected parameters are Ω_1_ = 0.037 *g*, Ω_2_ = 0.0775 *g*, and *J* = 20 *g*. We choose the experimental dissipative factors *γ* = 2.72 × 10^−4^ *g*, *κ* = 2.8 × 10^−2^ *g*, and *γ*_*ϕ*_ = 6.8 × 10^−5^ *g*. (**b**) Fidelity and purity of the steady state $|{{\rm{\Phi }}}_{1}^{0}\rangle$, plotted versus the atomic dephasing rate *γ*_*ϕ*_ with the experimental dissipative factors *γ* = 2.72 × 10^−4^ *g* and *κ* = 2.8 × 10^−2^ *g*. *γ*_*ϕ*_ varies from 0 to 1.2 × 10^−4^ *g* at time 8 × 10^3^/*g*.

Different from the atomic spontaneous emission, atoms are characterized by another dissipation factor: pure dephasing. This detrimental effect will be included in the numerical simulation. The Lindblad operator describing the dephasing of atom *n* can be written as ${\hat{L}}_{{\gamma }_{\phi }}^{n}=\sqrt{{\gamma }_{\phi }/2}(|{e}_{n}\rangle \langle {e}_{n}|-|{g}_{n}\rangle \langle {g}_{n}|)$ (*n* = 1, 2), where *γ*_*ϕ*_ is the atomic dephasing rate. We choose the pure dephasing time ${T}_{\phi }\simeq 36$ μs (${\gamma }_{\phi }=1/{T}_{\phi }\simeq 6.8\times {10}^{-5}\,g$), based on the experimental work in^37^, and solve the master equation numerically to qualify its influence on our scheme, as shown in Fig. 8(a). We plot the fidelity and purity of the target state $|{{\rm{\Phi }}}_{1}^{0}\rangle$ as functions of *gt* with the initial state |Φ_0_〉 by taking the optimized Rabi frequencies of the drivings as Ω_1_ = 0.037 *g* and Ω_2_ = 0.0775 *g*, the cavity-cavity hopping strength as *J* = 20 *g*, and the experimental dissipative factors as *κ* = 2.8 × 10^−2^ *g* and *γ* = 2.72 × 10^−4^ *g*. After comparing Figs 3 and 8(a), one can see that the dephasing has a slight influence on the fidelity and purity, which are approximately 0.7% and 1% lower than those without consideration of the effects of dephasing, respectively. In Fig. 8(b), we consider the system in stabilization (the evolution time *t* = 8 × 10^3^/*g*) and plot the fidelity and purity versus the atomic dephasing rate *γ*_*ϕ*_. The fidelity and purity are observed to decrease as *γ*_*ϕ*_ increases. When *γ*_*ϕ*_ changes from 0 to 1.2 × 10^−5^ *g*, the fidelity and purity are decreased only by 1.1% and 1.97%, respectively. This result occurs because atomic dephasing results in a population transfer between the singlet states |*ϕ*_−_〉 and |*ϕ*_+_〉, decreasing the fidelity and purity of the target steady state.

**Figure 9:**
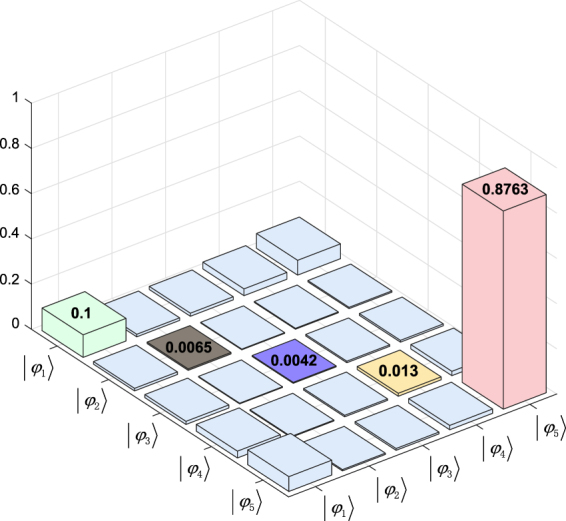
Truth table of the steady-state density matrix. The parameters used are *κ* = 2 × 2.8 × 10^−2^ *g* and *γ* = 2.72 × 10^−4^ *g*, and the optimized Rabi frequencies of the drivings are Ω_1_ = 0.037 *g* and Ω_2_ = 0.0775 *g*.

**Figure 10:**
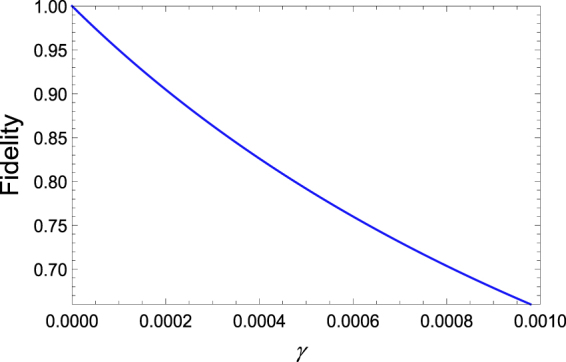
Fidelity of the target steady state as a function of the atomic spontaneous emission rate.

To further understand the proposed dissipative entanglement preparation scheme, we derive the stationary fidelity in analytical form for the target state with some reasonable approximations. First, we adiabatically eliminate the non-resonance coupling terms from Eq. (9) using the rotating wave approximation under the following conditions: the detuning Δ_*k*_ = *ω*_0_ − *ω*_*k*_ is equal to −(−1)^*k*^ *g* (*k* = 1, 2), and the weak excitation ${{\rm{\Omega }}}_{k}\ll g$. Second, there are two processes for generating the steady entanglement $|{{\rm{\Phi }}}_{0}\rangle \mathop{\leftrightarrow }\limits^{{{\rm{\Omega }}}_{1}}|{{\rm{\Phi }}}_{1}^{-}\rangle \mathop{\leftrightarrow }\limits^{{{\rm{\Omega }}}_{2}}|{{\rm{\Phi }}}_{2}^{0}\rangle \mathop{\to }\limits^{\kappa }|{{\rm{\Phi }}}_{1}^{0}\rangle$ and $|{{\rm{\Phi }}}_{0}\rangle \mathop{\leftrightarrow }\limits^{{{\rm{\Omega }}}_{2}}|{{\rm{\Phi }}}_{1}^{+}\rangle \mathop{\leftrightarrow }\limits^{{{\rm{\Omega }}}_{1}}|{{\rm{\Phi }}}_{2}^{0}\rangle \mathop{\to }\limits^{\kappa }|{{\rm{\Phi }}}_{1}^{0}\rangle$. To reduce the dimensions of the system, we incorporate the same dynamical processes by eliminating the intermediate state $|{{\rm{\Phi }}}_{1}^{+}\rangle$ because both processes could drive the initial state to $|{{\rm{\Phi }}}_{2}^{0}\rangle$ and then decay to $|{{\rm{\Phi }}}_{1}^{0}\rangle$. Therefore, we set |*φ*_1_〉 = |Φ_0_〉, $|{\varphi }_{2}\rangle =|{{\rm{\Phi }}}_{1}^{-}\rangle$, $|{\varphi }_{3}\rangle =|{{\rm{\Phi }}}_{2}^{0}\rangle$, $|{\varphi }_{4}\rangle =|{{\rm{\Phi }}}_{2}^{1}\rangle$, and $|{\varphi }_{5}\rangle =|{{\rm{\Phi }}}_{1}^{0}\rangle$, and we define *ρ*_*j*,*k*_(*t*) = 〈*φ*_*j*_|*ρ*(*t*)|*φ*_*k*_〉 (*j*,*k* = 1, 2, 3, 4, 5), where *ρ*(*t*) is the density operator of the system. Because the decays of states |*φ*_2_〉, |*φ*_3_〉, and |*φ*_4_〉 are dominated by dissipation of the bosonic modes, we can discard the effects of atomic spontaneous emission associated with these states. With this assumption, the probability of the system being in a different state is governed by the stationary state equation *dρ*_*j*,*k*_(*t*)/*dt* = 0. By analytically solving the Lindblad master equation, we can obtain the diagonal elements of the five-dimensional density matrix (see the Methods subsection for the detailed calculation). Because the 25 stationary state equations are algebraic equations, we do not need to introduce a preset initial state, in contrast to the differential equations, which require presetting a certain initial state. Therefore, the stationary solutions have a generality in indirectly verifying the uniqueness of $|{{\rm{\Phi }}}_{1}^{0}\rangle$, i.e., arbitrary initial states can be driven to the target state $|{{\rm{\Phi }}}_{1}^{0}\rangle$, which leads to the target state having an identical fidelity. This is in agreement with the numerical simulation in Fig. 4. To assess the accuracy of the approximate analytical solution, we take the experimental parameters *κ* = 2 × 2.8 × 10^−2^ *g* and *γ* = 2.72 × 10^−4^ *g* and the optimized Rabi frequencies of the drivings Ω_1_ = 0.037 *g* and Ω_2_ = 0.0775 *g*. Here, *κ* is the collective bosonic mode decay; it is reasonable to select a value of twice the leakage of the cavity field. In Fig. 9, we present a truth table of the steady-state density matrix constructed corresponding to the analytical results; the table indicates that the fidelity of the target steady state is approximately *ρ*_5,5_ = 0.8763. This is only slightly different from the numerical fidelity of 0.889 for the initial state |Φ_0_〉. Note that the approximation is valid under the condition that spontaneous emission is a passive source in driving the atomic qubits out of the target steady state. In Fig. 10, the fidelity of the target steady state is plotted as a function of the atomic spontaneous emission rate *γ*. The result shows that the fidelity decreases as the atomic spontaneous emission rate increases to a bearable extent; further increases in the dissipative factors will have greater negative effects on the performance of the scheme. In addition, to analytically verify the stationarity of the target entangled state $|{{\rm{\Phi }}}_{1}^{0}\rangle$, we consider an ideal (the spontaneous emission rate *γ* = 0) and effective $(|{{\rm{\Phi }}}_{0}\rangle \mathop{\leftrightarrow }\limits^{{\rm{\Omega }}}|{{\rm{\Phi }}}_{2}^{0}\rangle \mathop{\to }\limits^{\kappa }|{{\rm{\Phi }}}_{1}^{0}\rangle )$ process. At this point, the density operator *ρ*(*t*) of the system becomes 3 × 3. The initial state of the system is assumed to be |Φ_0_〉; thus, we can analytically give

13 $$d{\rho }_{\mathrm{5,5}}(t)/dt=\frac{16{e}^{-\frac{t\kappa }{2}}{{\rm{\Omega }}}^{2}\kappa \,{\mathrm{Sinh}}^{2}(\tfrac{1}{4}t\sqrt{{\kappa }^{2}-16{{\rm{\Omega }}}^{2}})}{{\kappa }^{2}-16{{\rm{\Omega }}}^{2}},$$

and

14 $$\begin{array}{rcl}{\rho }_{\mathrm{5,5}}(t) & = & \frac{1}{\mathrm{2(}Q{)}^{\mathrm{3/2}}}{e}^{-\frac{t\kappa }{2}}[2{e}^{\frac{t\kappa }{2}}{(Q)}^{\mathrm{3/2}}+{e}^{-\frac{\sqrt{Q}t}{2}}\kappa (Q-\sqrt{Q}\kappa )\\  &  & +32\sqrt{Q}{{\rm{\Omega }}}^{2}-{e}^{\frac{\sqrt{Q}t}{2}}\kappa [\kappa (\sqrt{Q}+\kappa )-16{{\rm{\Omega }}}^{2}]],\end{array}$$

**Figure 11:**
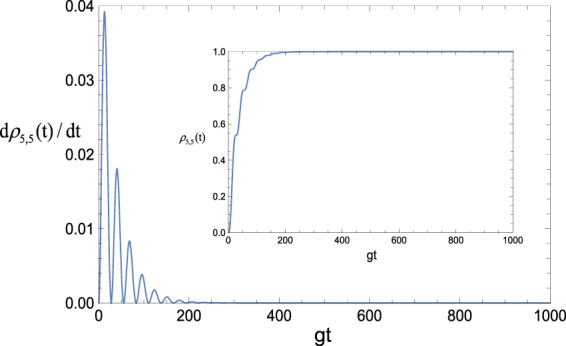
Variation rate of the probability for the target state versus *gt* with the initial state |Φ_0_〉. The chosen parameter is Ω = Ω_1_ + Ω_2_, in which Ω_1_ = 0.037 *g* and Ω_2_ = 0.0775 *g*. The selected dissipative factor is *κ* = 2 × 2.8 × 10^−2^ *g*. The inset shows the probability of the target state as a function of *gt*.

*Q* = *κ* ^2^ − 16Ω^2^ and Ω reprwhere the parameteresents the effective Rabi frequency that directly drives the transition between |Φ_0_〉 and $|{{\rm{\Phi }}}_{2}^{0}\rangle$. In Fig. 11, we plot the time evolution of *dρ*_5,5_(*t*)/*dt* with Eq. (13) by taking the parameter Ω = Ω_1_ + Ω_2_ = 0.1145 *g* and the dissipative factor *κ* = 2 × 2.8 × 10^−2^ *g*, which exhibits a oscillatory behavior before *dρ*_5,5_(*t*)/*dt* approaches 0 as the interaction time increases. This is the result of the competition between the coherent driving and dissipation. *dρ*_5,5_(*t*)/*dt* = 0 means that the probability of the target state reaching stabilization is time invariant. We also plot the probability of the target state versus *gt* in the inset of Fig. 11, which shows a very good agreement with the variance of *dρ*_5,5_(*t*)/*dt*.

**Figure 12:**
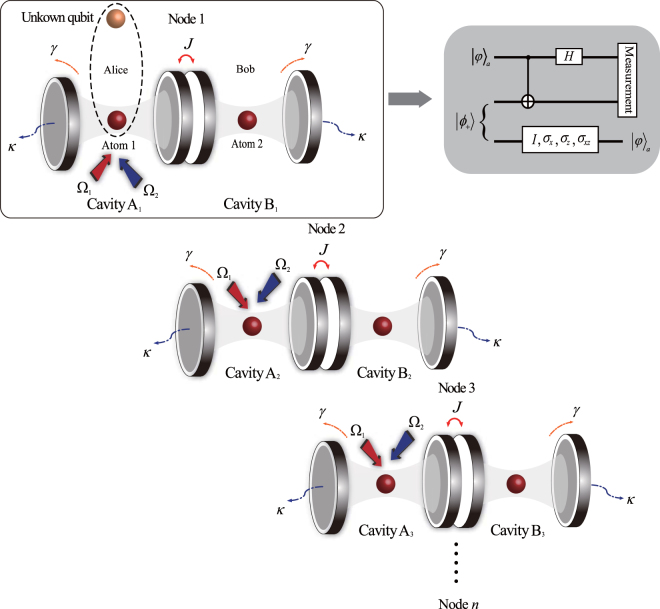
Schematic diagram for the implementation of quantum state transfer using a standard teleportation procedure. The information of the unknown qubit can be transferred from the first node to the *n*th node. The solid box denotes the first node to teleport an unknown quantum state from Alice to Bob. The dashed box in the first panel represents two qubits that belong to the same participant. The gray box to the upper right is a quantum circuit of teleportation for the first node. Here, *H* represents a Hadamard operation, *σ*_*x*_ and *σ*_*z*_ are the Pauli operators representing local qubit-flip operations, and *I* is the identity operator.

As a practical application of our dissipative entanglement preparation scheme in quantum communication, we construct a quantum state transfer setup with multiple nodes, as shown in Fig. 12. Suppose that each node is initially prepared in the entangled steady state |*ϕ*_+_〉, which is shared by the sender (Alice) and the receiver (Bob). The unknown quantum state (referred to as a) to be transferred in Alice’s hands is |*φ*〉_*a*_ = *α*|0〉_*a*_ + *β*|1〉_*a*_, where *α* and *β* are unknown parameters, with |*α*|^2^ + |*β*|^2^ = 1. Using a standard teleportation procedure^56,57^, Bob can deterministically recover the unknown state only by applying some local operations (*I*_2_, ${\sigma }_{{x}_{2}}$, ${\sigma }_{{z}_{2}}$, and ${\sigma }_{{x}_{2}}{\sigma }_{{z}_{2}}$) to atom 2. In the following, the atomic state at Bob’s side is an unknown quantum state, which can be subsequently transferred from the first node to the *n*th node by performing the same operation.

In summary, we have proposed a scheme for producing and stabilizing a Bell state in a coupled cavity system via cavity decay. The distinct feature of our scheme is that only one atom needs to be driven by classical control fields. This not only greatly simplifies the experimental implementation between separate nodes of a quantum network but also makes the scheme robust against drive amplitude fluctuations and cavity field decay. Using the presently available experimental parameters, a steady Bell state with a high fidelity and purity can be obtained by optimizing the driving amplitudes. We have also given an analytical solution for the stationary fidelity to demonstrate the validity of our scheme, which is highly consistent with the numerical solution because we simply consider the five-dimensional subspace in the dressed state picture. Furthermore, as a practical application, we have constructed a quantum state transfer scheme with multiple nodes based our basic model. The above analysis and numerical simulations show that the present scheme is feasible with the current technology and is generally suitable for different qubit-resonator systems.

## Methods

We express the density operator *ρ*(*t*) of the system using the five basis states |*φ*_1_〉 = |Φ_0_〉, $|{\varphi }_{2}\rangle =|{{\rm{\Phi }}}_{1}^{-}\rangle$, $|{\varphi }_{3}\rangle =|{{\rm{\Phi }}}_{2}^{0}\rangle$, $|{\varphi }_{4}\rangle =|{{\rm{\Phi }}}_{2}^{1}\rangle$, and $|{\varphi }_{5}\rangle =|{{\rm{\Phi }}}_{1}^{0}\rangle$ and assume that the state of the system at arbitrary time *t* is |*ψ*(*t*)〉 = *c*_1_(*t*)|*φ*_1_〉 + *c*_2_(*t*)|*φ*_2_〉 + *c*_3_(*t*)|*φ*_3_〉 + *c*_4_(*t*)|*φ*_4_〉 + *c*_5_(*t*)|*φ*_5_〉, where *c*_*i*_ (*i* = 1, 2, 3, 4, 5) denotes the probability amplitudes for the corresponding states. Hence, the density operator of the system in matrix form is given by

15 $$\rho (t)=[\begin{array}{ccccc}|{c}_{1}(t{)|}^{2} & {c}_{1}(t){c}_{2}^{\ast }(t) & {c}_{1}(t){c}_{3}^{\ast }(t) & {c}_{1}(t){c}_{4}^{\ast }(t) & {c}_{1}(t){c}_{5}^{\ast }(t)\\ {c}_{2}(t){c}_{1}^{\ast }(t) & |{c}_{2}(t{)|}^{2} & {c}_{2}(t){c}_{3}^{\ast }(t) & {c}_{2}(t){c}_{4}^{\ast }(t) & {c}_{2}(t){c}_{5}^{\ast }(t)\\ {c}_{3}(t){c}_{1}^{\ast }(t) & {c}_{3}(t){c}_{2}^{\ast }(t) & |{c}_{3}(t{)|}^{3} & {c}_{3}(t){c}_{4}^{\ast }(t) & {c}_{3}(t){c}_{5}^{\ast }(t)\\ {c}_{4}(t){c}_{1}^{\ast }(t) & {c}_{4}(t){c}_{2}^{\ast }(t) & {c}_{4}(t){c}_{3}^{\ast }(t) & |{c}_{4}(t{)|}^{2} & {c}_{4}(t){c}_{5}^{\ast }(t)\\ {c}_{5}(t){c}_{1}^{\ast }(t) & {c}_{5}(t){c}_{2}^{\ast }(t) & {c}_{5}(t){c}_{3}^{\ast }(t) & {c}_{5}(t){c}_{4}^{\ast }(t) & |{c}_{5}(t{)|}^{2}\end{array}],$$

in which the diagonal elements represent the probability, also known as the fidelity, of the corresponding states. We define ${\rho }_{j,k}(t)={c}_{j}(t){c}_{k}^{\ast }(t)$ (*j*, *k* = 1, 2, 3, 4, 5). The evolution of the system is described by the following coupled differential equations for the corresponding density matrix elements:

16 $$\begin{array}{rcl}d{\rho }_{\mathrm{1,1}}(t)/dt & = & \frac{1}{2}[i{{\rm{\Omega }}}_{1}({\rho }_{\mathrm{1,2}}(t)-{\rho }_{\mathrm{2,1}}(t))+\kappa {\rho }_{\mathrm{2,2}}(t)+2\gamma {\rho }_{\mathrm{5,5}}(t)],\\ d{\rho }_{\mathrm{1,2}}(t)/dt & = & -\frac{1}{4}\kappa {\rho }_{\mathrm{1,2}}(t)-\frac{1}{2}i{{\rm{\Omega }}}_{2}({\rho }_{\mathrm{1,3}}(t)+\frac{\sqrt{6}}{3}{\rho }_{\mathrm{1,4}}(t))\\  &  & +\frac{1}{2}i{{\rm{\Omega }}}_{1}({\rho }_{\mathrm{1,1}}(t)-{\rho }_{\mathrm{2,2}}(t))+\frac{\sqrt{6}}{6}\kappa {\rho }_{\mathrm{2,4}}(t),\\ d{\rho }_{\mathrm{1,3}}(t)/dt & = & -\frac{1}{2}i({{\rm{\Omega }}}_{2}{\rho }_{\mathrm{1,2}}(t)-i\kappa {\rho }_{\mathrm{1,3}}(t)+{{\rm{\Omega }}}_{1}{\rho }_{\mathrm{2,3}}(t)),\\ d{\rho }_{\mathrm{1,4}}(t)/dt & = & -\frac{1}{6}i(\sqrt{6}{{\rm{\Omega }}}_{2}{\rho }_{\mathrm{1,2}}(t)-i\kappa {\rho }_{\mathrm{1,4}}(t)+3{{\rm{\Omega }}}_{1}{\rho }_{\mathrm{2,4}}(t)),\\ d{\rho }_{\mathrm{1,5}}(t)/dt & = & -\frac{1}{2}\gamma {\rho }_{\mathrm{1,5}}(t)-\frac{\sqrt{2}}{2}\kappa {\rho }_{\mathrm{2,3}}(t)-\frac{1}{2}i{{\rm{\Omega }}}_{1}{\rho }_{\mathrm{2,5}}(t),\\ d{\rho }_{\mathrm{2,2}}(t)/dt & = & -\frac{1}{2}i{{\rm{\Omega }}}_{1}({\rho }_{\mathrm{1,2}}(t)-{\rho }_{\mathrm{2,1}}(t))-\kappa \mathrm{(3}{\rho }_{\mathrm{2,2}}(t)-2{\rho }_{\mathrm{4,4}}(t))\\  &  & -\frac{1}{6}i{{\rm{\Omega }}}_{2}(3{\rho }_{\mathrm{2,3}}(t)+\sqrt{6}{\rho }_{\mathrm{2,4}}(t)-3{\rho }_{\mathrm{3,2}}(t)-\sqrt{6}{\rho }_{\mathrm{4,2}}(t)),\end{array}$$

$$\begin{array}{rcl}d{\rho }_{\mathrm{2,3}}(t)/dt & = & -\frac{1}{2}i{{\rm{\Omega }}}_{1}{\rho }_{\mathrm{1,3}}(t)-\frac{3}{4}\kappa {\rho }_{\mathrm{2,3}}(t)\\  &  & -\frac{1}{6}i{{\rm{\Omega }}}_{2}(3{\rho }_{\mathrm{2,2}}(t)-3{\rho }_{\mathrm{3,3}}(t)-\sqrt{6}{\rho }_{\mathrm{4,3}}(t)),\\ d{\rho }_{\mathrm{2,4}}(t)/dt & = & -\frac{1}{2}i{{\rm{\Omega }}}_{1}{\rho }_{\mathrm{1,4}}(t)-\frac{5}{12}\kappa {\rho }_{\mathrm{2,4}}(t)\\  &  & -\frac{1}{6}i{{\rm{\Omega }}}_{2}(\sqrt{6}{\rho }_{\mathrm{2,2}}(t)-3{\rho }_{\mathrm{3,4}}(t)-\sqrt{6}{\rho }_{\mathrm{4,4}}(t)),\\ d{\rho }_{\mathrm{2,5}}(t)/dt & = & -\frac{1}{2}i{{\rm{\Omega }}}_{1}{\rho }_{\mathrm{1,5}}(t)-\kappa (\frac{1}{4}{\rho }_{\mathrm{2,5}}(t)+\frac{\sqrt{3}}{3}{\rho }_{\mathrm{4,3}}(t))\\  &  & +i{{\rm{\Omega }}}_{2}(\frac{1}{2}{\rho }_{\mathrm{3,5}}(t)+\frac{\sqrt{6}}{6}{\rho }_{\mathrm{4,5}}(t)),\\ d{\rho }_{\mathrm{3,3}}(t)/dt & = & \frac{1}{2}i{{\rm{\Omega }}}_{2}({\rho }_{\mathrm{2,3}}(t)-{\rho }_{\mathrm{3,2}}(t))-\kappa {\rho }_{\mathrm{3,3}}(t),\\ d{\rho }_{\mathrm{3,4}}(t)/dt & = & i{{\rm{\Omega }}}_{2}(\frac{1}{2}{\rho }_{\mathrm{2,4}}(t)-\frac{\sqrt{6}}{6}{\rho }_{\mathrm{3,2}}(t))-\frac{2}{3}\kappa {\rho }_{\mathrm{3,4}}(t),\\ d{\rho }_{\mathrm{3,5}}(t)/dt & = & \frac{1}{2}i{{\rm{\Omega }}}_{2}{\rho }_{\mathrm{2,5}}(t)-\frac{1}{2}\kappa {\rho }_{\mathrm{3,5}}(t),\\ d{\rho }_{\mathrm{4,4}}(t)/dt & = & \frac{\sqrt{6}}{6}i{{\rm{\Omega }}}_{2}({\rho }_{\mathrm{2,4}}(t)-{\rho }_{\mathrm{4,2}}(t))-\frac{1}{3}\kappa {\rho }_{\mathrm{4,4}}(t),\\ d{\rho }_{\mathrm{4,5}}(t)/dt & = & \frac{1}{6}(i{{\rm{\Omega }}}_{2}\sqrt{6}{\rho }_{\mathrm{2,5}}(t)-\kappa {\rho }_{\mathrm{4,5}}(t)),\\ d{\rho }_{\mathrm{5,5}}(t)/dt & = & \kappa {\rho }_{\mathrm{3,3}}(t)-\gamma {\rho }_{\mathrm{5,5}}(t),\end{array}$$

in which we have omitted the corresponding transposed-conjugate terms because *dρ*_*j*,*k*_/*dt* = [*dρ*_*k*,*j*_/*dt*]^†^ (*k* ≠ *j*). The analytical solutions for the stationary state equation *dρ*_*j*,*k*_/*dt* = 0 can be obtained as follows:

17 $$\begin{array}{rcl}{\rho }_{\mathrm{1,1}} & = & \frac{\gamma }{M}[288{{\rm{\Omega }}}_{1}^{6}+160{\kappa }^{2}{{\rm{\Omega }}}_{1}^{3}{{\rm{\Omega }}}_{2}+8{{\rm{\Omega }}}_{1}^{4}\mathrm{(73}{\kappa }^{2}-9{{\rm{\Omega }}}_{2}^{2})\\  &  & +8{{\rm{\Omega }}}_{1}\mathrm{(30}{\kappa }^{4}{{\rm{\Omega }}}_{2}+37{\kappa }^{2}{{\rm{\Omega }}}_{2}^{3})\\  &  & +({\kappa }^{2}+6{{\rm{\Omega }}}_{2}^{2})\,\mathrm{(30}{\kappa }^{4}+169{\kappa }^{2}{{\rm{\Omega }}}_{2}^{2}+198{{\rm{\Omega }}}_{2}^{4})\\  &  & +{{\rm{\Omega }}}_{1}^{2}\mathrm{(248}{\kappa }^{4}+682{\kappa }^{2}{{\rm{\Omega }}}_{2}^{2}+984{{\rm{\Omega }}}_{2}^{4})],\\ {\rho }_{\mathrm{2,2}} & = & \frac{4\gamma {{\rm{\Omega }}}_{1}^{2}}{M}\mathrm{[30}{\kappa }^{4}+72{{\rm{\Omega }}}_{1}^{4}+109{\kappa }^{2}{{\rm{\Omega }}}_{2}^{2}\\  &  & +60{{\rm{\Omega }}}_{2}^{4}+2{{\rm{\Omega }}}_{1}^{2}\mathrm{(64}{\kappa }^{2}+51{{\rm{\Omega }}}_{2}^{2})],\\ {\rho }_{\mathrm{3,3}} & = & \frac{12\gamma {{\rm{\Omega }}}_{1}^{2}{{\rm{\Omega }}}_{2}^{2}}{M}\mathrm{(10}{\kappa }^{2}+36{{\rm{\Omega }}}_{1}^{2}+23{{\rm{\Omega }}}_{2}^{2}),\\ {\rho }_{\mathrm{4,4}} & = & \frac{24\gamma {{\rm{\Omega }}}_{1}^{2}{{\rm{\Omega }}}_{2}^{2}}{M}\mathrm{(30}{\kappa }^{2}+20{{\rm{\Omega }}}_{1}^{2}+37{{\rm{\Omega }}}_{2}^{2}),\\ {\rho }_{\mathrm{5,5}} & = & \frac{12\kappa {{\rm{\Omega }}}_{1}^{2}{{\rm{\Omega }}}_{2}^{2}}{M}\mathrm{(10}{\kappa }^{2}+36{{\rm{\Omega }}}_{1}^{2}+23{{\rm{\Omega }}}_{2}^{2}),\end{array}$$

where the parameter

18 $$\begin{array}{rcl}M & = & 576\gamma {{\rm{\Omega }}}_{1}^{6}+160\gamma {\kappa }^{2}{{\rm{\Omega }}}_{1}^{3}{{\rm{\Omega }}}_{2}+8\gamma {\kappa }^{2}{{\rm{\Omega }}}_{1}{{\rm{\Omega }}}_{2}\mathrm{(30}{\kappa }^{2}+37{{\rm{\Omega }}}_{2}^{2})\\  &  & \times 8{{\rm{\Omega }}}_{1}^{4}\mathrm{[137}\gamma {\kappa }^{2}+6{{\rm{\Omega }}}_{2}^{2}\mathrm{(26}\gamma +9\kappa )]\\  &  & +\gamma ({\kappa }^{2}+6{{\rm{\Omega }}}_{2}^{2})\,\mathrm{(30}{\kappa }^{4}+169{\kappa }_{2}{{\rm{\Omega }}}_{2}^{2}+198{{\rm{\Omega }}}_{2}^{4})\\  &  & \times 2{{\rm{\Omega }}}_{1}^{2}[184\gamma {\kappa }^{4}+{\kappa }^{2}{{\rm{\Omega }}}_{2}^{2}\mathrm{(979}\gamma +60\kappa )+6{{\rm{\Omega }}}_{2}^{4}\mathrm{(199}\gamma +23\kappa )].\end{array}$$
